# The Role of *Henosepilachna vigintioctopunctata* in Facilitating the Spread of Tomato Brown Rugose Fruit Virus (ToBRFV) Among Hosts

**DOI:** 10.3390/insects16121225

**Published:** 2025-12-03

**Authors:** Xing-Xing Wang, Qing-Jiang Xing, Chong Zhang, Ya-Nan Liu, Tong-Xian Liu, Yi Zhang

**Affiliations:** 1 Shandong Engineering Research Center for Environment-Friendly Agricultural Pest Management, College of Plant Health and Medicine, Qingdao Agricultural University, Qingdao 266109, China; wxx0521@qau.edu.cn (X.-X.W.); xingqj2000@stu.qau.edu.cn (Q.-J.X.); 18209829603@163.com (C.Z.); lyn2001@stu.qau.edu.cn (Y.-N.L.); 2 Institute of Entomology, Guizhou University, Guiyang 550025, China; tx.liu@gzu.edu.cn

**Keywords:** ToBRFV, *Henosepilachna vigintioctopunctata*, virus-vector

## Abstract

Tomato brown rugose fruit virus (ToBRFV), a highly stable and mechanically transmissible tobamovirus, poses a significant threat to solanaceous crops worldwide, particularly tomato (*Solanum lycopersicum*). While its transmission via human activities and contaminated materials is well-documented, the role of common phytophagous insects in its epidemiology remains less understood. *Henosepilachna vigintioctopunctata*, the Hadda beetle, is a common pest of Solanaceae with a host range that overlaps extensively with that of ToBRFV. This study aimed to quantify the beetle’s capacity to act as a mechanical vector and to assess its potential epidemiological impact. Using reverse transcription quantitative PCR (RT-qPCR), we evaluated beetle-mediated transmission efficiency, the persistence of its virus-carrying capacity, and its ability to vector the virus to various solanaceous hosts. Our results demonstrate that *H. vigintioctopunctata* efficiently acquires and transmits ToBRFV to tomato and other key hosts, including black nightshade (*S. nigrum*), pepper (*Capsicum annuum*), and eggplant (*Solanum melongena*). The virus was retained and remained transmissible by beetles for up to 48 h post-acquisition, providing a significant window for dispersal. Viral particles were most abundant in the digestive tract, consistent with ingestion of infected tissue, and declined rapidly on external body parts, confirming a non-circulative, mechanical transmission mechanism. Furthermore, feeding wounds created by non-viruliferous beetles increased plant susceptibility to subsequent infection from environmental contamination. We conclude that *H. vigintioctopunctata* acts as a potential mechanical vector that might amplify ToBRFV spread at local and landscape levels. This highlights a synergistic interaction between a native pest and an invasive pathogen, underscoring the necessity of incorporating beetle management into integrated strategies for controlling ToBRFV.

## Simple Summary

This study demonstrates that the hadda beetle (*Henosepilachna vigintioctopunctata*) serves as an effective mechanical vector for Tomato brown rugose fruit virus (ToBRFV), a highly contagious and damaging pathogen of solanaceous crops. Through quantitative transmission assays, we confirmed that the beetle efficiently acquires ToBRFV from infected tomato plants and transmits it to key hosts, including pepper, eggplant, and black nightshade. The virus remains transmissible for up to 48 h post-acquisition, with the digestive tract identified as the primary site of virus retention, consistent with a non-circulative transmission mechanism. Furthermore, feeding damage by non-viruliferous beetles significantly enhances plant susceptibility to virus infection. Cage experiments revealed that beetle activity substantially amplifies virus spread across mixed host populations. These findings elucidate a synergistic interaction between an indigenous pest and an emerging viral pathogen, underscoring the necessity of incorporating vector management into integrated strategies for controlling ToBRFV epidemics in agricultural systems.

## 1. Introduction

Tomato *Solanum lycopersicum*, as a globally important economic crop, is highly susceptible to pests and diseases, which severely impact its quality and yield. In recent years, tomato brown rugose fruit virus (ToBRFV) has emerged as a critical pathogen of solanaceous crops, characterized by its rapid spread, high infectivity, and severe damage, posing a major threat to the global tomato industry [1]. Concurrently, the Hadda beetle (*Henosepilachna vigintioctopunctata*), a pest known for feeding on a wide range of solanaceous plants, is frequently found in tomato fields, especially under intensive cultivation [2,3]. It is reported to be a major pest of solanaceous crops, primarily targeting *Solanum* species, including tomato, but also affecting plants in the *Capsicum* and *Physalis* genera [2,4]. Predominantly distributed across East and Southeast Asia, the pest has expanded to Australia and Central Asia [5,6]. Therefore, investigating whether an interaction exists between these two biological threats that could exacerbate their combined damage is of significant scientific and practical importance.

ToBRFV belongs to the genus *Tobamovirus* within the family *Virgaviridae.* It is a positive-sense single-stranded RNA virus with a genome of approximately 6.4 kb that encodes four open reading frames. The viral particles are rigid rods, about 300 nm in length and 18 nm in diameter [7]. The virus has caused substantial damage to solanaceous crops, including tomatoes [8,9], peppers, eggplants, and various solanaceous weeds, with particularly severe impacts on high-value horticultural crops [10,11]. In tomato plants, ToBRFV infection leads to symptoms such as interveinal chlorosis, leaf deformation, mosaic patterns, and discoloration. Infected fruits exhibit uneven ripening, malformation, and necrosis, all of which severely reduce crop yield and marketability [1].

The ability of ToBRFV to persist and be transmitted among various solanaceous crops and weeds poses a significant challenge for disease management. Different solanaceous species exhibit varied susceptibility and symptomology; however, the cross-species transmissibility of ToBRFV allows it to persist in cropping systems and affect subsequent plantings [12,13,14].

ToBRFV is highly contagious and is primarily transmitted through mechanical contact. Human agricultural practices, such as plant handling, can inadvertently spread the virus, and plant wounds significantly increase the risk of infection [12]. Long-distance transmission occurs mainly through contaminated seeds and seedlings. Although viral particles have been detected on the seed coat, they have not been found in the embryo [15]. Secondary and generational transmission in the field often occurs through infected plant debris in the soil, which can spread the virus to newly planted crops. Additionally, irrigation and fertilization practices can amplify the virus’s spread within agricultural fields [16]. Therefore, a thorough understanding of the virus’s transmission dynamics between crops and weeds at different growth stages is crucial for developing systematic control measures.

Research on insect-mediated transmission of ToBRFV is still limited. While certain hemipteran insects, particularly whiteflies, are established vectors for tomato viruses [17], there is evidence that bumblebees and the tomato leafminer (*Tuta absoluta*) can also effectively transmit ToBRFV. These insects primarily feed on solanaceous plants or participate in pollination processes, transmitting the virus through mechanical friction, with no direct evidence that ToBRFV replicates within the insect body [18,19]. Despite the limited evidence for insect-assisted transmission, the dispersal capabilities of insects pose a significant risk to solanaceous crops, especially in facilitating the spread of the virus to weeds, which complicates comprehensive control efforts.

As a phytophagous beetle, the feeding range of *H. vigintioctopunctata* substantially overlaps with that of ToBRFV hosts [2,4]. With its strong flight capabilities, long lifespan, and efficient host-location ability [4], *H. vigintioctopunctata* could potentially facilitate ToBRFV transmission across distant fields and among diverse solanaceous hosts, including both crops and weeds. Moreover, the beetle’s feeding behavior is particularly damaging to leaf tissues, creating large wounds that expose internal tissues. Unlike leaf beetles that merely puncture leaves, *H. vigintioctopunctata* skeletonizes the leaf tissue while preserving the opposite epidermis, creating a characteristic windowpane feeding pattern. This feeding method significantly increases the risk of viral infection, even when the beetle itself is virus-free [4,12]. Previous studies have documented that *H. vigintioctopunctata* can facilitate virus spread through feeding on host plants [20,21]. However, whether it plays a role in the transmission of ToBRFV remains unknown.

This research was designed to address the potential risk by systematically evaluating the role of *H. vigintioctopunctata* in the ToBRFV disease cycle. The specific objectives were: (1) to confirm and quantify the efficiency of beetle-mediated mechanical transmission of ToBRFV to tomato; (2) to determine the retention period of infectivity in the beetle, a key parameter for dispersal potential; (3) to assess the beetle’s capacity to transmit the virus to other important solanaceous hosts; and (4) to evaluate the indirect role of beetle feeding wounds in increasing plant susceptibility to infection.

## 2. Materials and Methods

### 2.1. Plants and Insects

Hadda beetles *H. vigintioctopunctata*, originally collected from *Solanum nigrum* in Chengyang District, Qingdao, Shandong Province, China, were reared in the laboratory for over one year (about 10 generations) on tomatoes (*S. lycopersicum*, cv. Zhongza9) and potatoes. In this study, all beetles were fed on tomatoes.

The tomato brown rugose fruit virus (ToBRFV) was collected from infected tomato plants in Pingdu, Shandong Province, China. Experimental plants were divided into two groups based on ToBRFV infection status. All plants were maintained in isolated conditions to prevent cross-contamination.

### 2.2. Virus Inoculation and Quantification

#### 2.2.1. Mechanical Inoculation (Positive Control)

To prepare infectious sap, leaves from systemically ToBRFV-infected tomato plants were homogenized in a Phosphate-Buffered Solution (PBS, pH 7.2). The homogenate was centrifuged at 12,000 rpm for 5 min, and the supernatant was used as the inoculum. For positive control treatments, young leaves of healthy plants were lightly dusted with 325-mesh quartz sand and then gently rubbed with a cotton swab dipped in the infectious sap.

#### 2.2.2. RNA Extraction and RT-qPCR

Total RNA was extracted from plant leaf discs (1 cm × 1 cm) or dissected beetle tissues using RNAiso Plus (Takara, Tokyo, Japan). First-strand cDNA was synthesized using the PrimeScript™ RT reagent Kit with gDNA Eraser (Takara, Tokyo, Japan). RT-qPCR was performed using SYBR^®^ Premix Ex Taq™ II (Takara, Tokyo, Japan) on an iQ5 Real-Time PCR Detection System (Bio-Rad, Berkeley, CA, USA). The PCR cycling conditions were: initial denaturation at 95 °C for 30 s, followed by 40 cycles of 95 °C for 5 s and 60 °C for 30 s. Primer sequences are listed in Supplementary Table S1.

#### 2.2.3. Quantification Method

Relative virus load was estimated using the 2^−ΔΔCt^ method, normalized against the *SlUBI* (*ubiquitin* for tomato) [22,23] or *HvRPL13* (*ribosomal protein*, *large subunit 13* for beetle tissues) [24] reference genes. For cross-species plant comparisons where reference genes differed, raw Cycle threshold (Ct) values were compared.

### 2.3. Beetle-Mediated Transmission Assays

#### 2.3.1. Virus Acquisition by Beetles

Adult beetles were starved for 12 h and then allowed to feed for 24 h on intact, systemically infected tomato plants (confirmed by RT-qPCR). This method was used to prepare viruliferous beetles for all subsequent experiments to simulate natural acquisition.

#### 2.3.2. Transmission to Tomato (*S. lycopersicum*)

**Figure 1:**
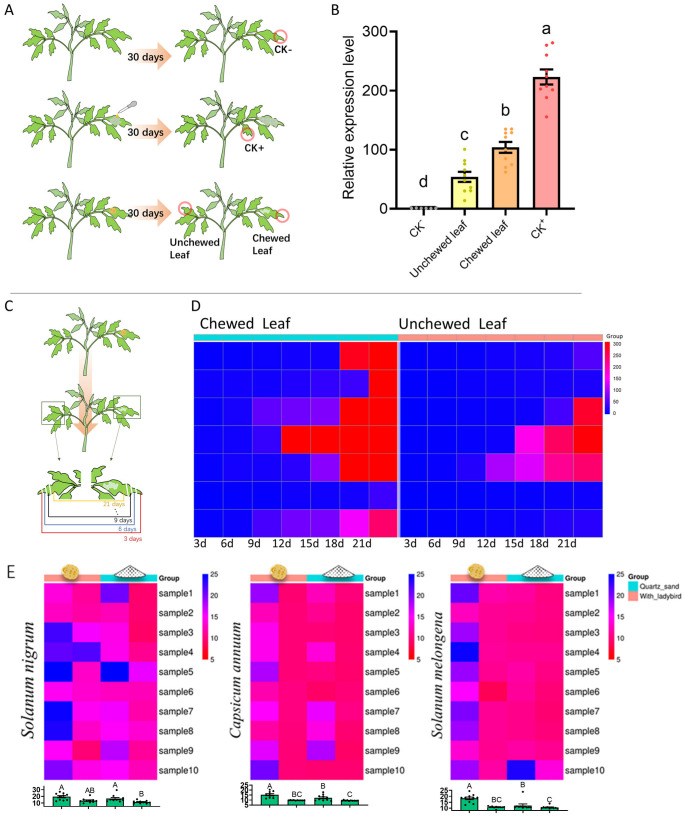
Validation of *H. vigintioctopunctata* transmission of ToBRFV and viral accumulation post-transmission. (**A**) Schematic of the three experimental treatments for testing virus transmission in tomato: Negative Control (CK−), Positive Control (CK+) using quartz sand abrasion, and Beetle-mediated transmission. (**B**) Relative ToBRFV expression (2^−ΔΔCt^) in tomato leaves 30 days post-inoculation. “Chewed leaf” refers to the leaf directly fed upon by a viruliferous beetle. “Unchewed leaf” refers to an unfed upper leaf from the same plant. (**C**) Schematic of the sampling strategy for the time-course experiment. (**D**) Heatmap visualizing temporal changes in virus load (based on RT-qPCR Ct values) in chewed and systemic leaves over 21 days. Lower Ct values (red) indicate higher virus loads. (**E**) Heatmaps and bar charts showing virus transmission from infected tomato to pepper (*C. annuum*), eggplant (*S. melongena*), and black nightshade (*S. nigrum*) at 15 and 30 days post-inoculation; The diagram of the ladybird and the quartz sand represents two different methods used in the experiment. Bars represent mean values ± standard error (SE). Different letters above bars indicate statistically significant differences (One-way ANOVA, Duncan’s test, *p* < 0.05).

One viruliferous beetle was placed on a single leaf of a healthy 4-week-old tomato plant and enclosed in a 10 cm × 12 cm mesh bag with one leaf. After the beetle had consumed approximately one-third of the leaf area, it was removed. The plants were then maintained in isolation for 30 days. Samples from the chewed leaf and an intact upper leaf were collected for RT-qPCR analysis of local and systemic infection, respectively. The experiment was performed with 10 replicate plants (Figure 1A).

#### 2.3.3. Temporal Dynamics of Infection

To monitor the progression of infection, an identical experiment was set up with 7 replicate plants. Leaf samples were collected from the chewed leaf and an opposite unchewed leaf every three days for a total of 21 days (Figure 1C).

### 2.4. Transmission to Other Solanaceous Hosts

#### 2.4.1. Non-Choice Assay

Adult beetles were starved for 12 h and then allowed to feed on infected tomato plants for 24 h. Viruliferous beetles were then transferred and enclosed in a 10 cm × 12 cm mesh bag with one leaf of individual healthy plants of pepper *C. annuum*, eggplant *S. melongena*, and black nightshade *S. nigrum* (N = 10 replicates per species). Beetles were allowed to feed for 24 h before being removed. Plants were grown for 30 days, and samples from systemic, undamaged leaves were collected at 15 and 30 days post-inoculation (dpi) for RT-qPCR analysis.

#### 2.4.2. Free-Choice Cage Experiment

**Figure 2:**
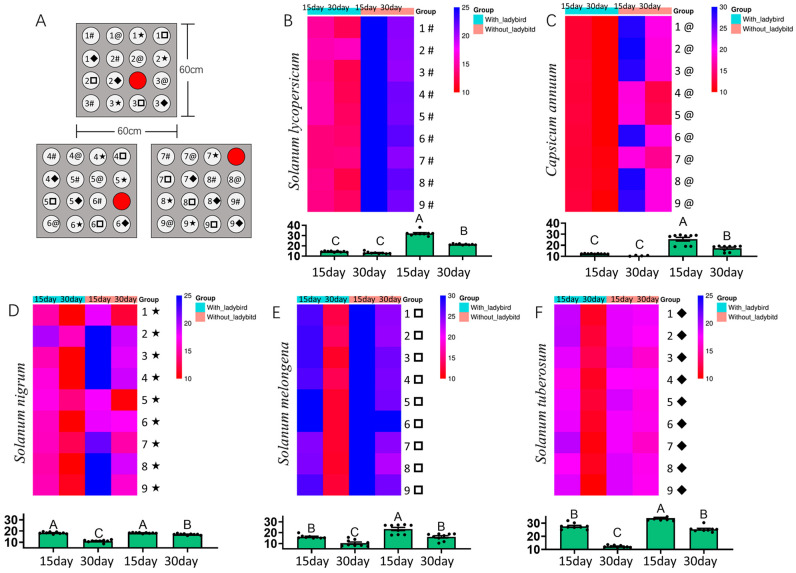
Outdoor cage experiment evaluating the role of *H. vigintioctopunctata* in amplifying ToBRFV transmission among mixed solanaceous hosts. (**A**) Schematic of the planting arrangement within the cage, we use five distinct symbols to represent five species of solanaceous plants (# for *S. lycopersicum*; @ for *C. annuum*; ★ for *S. nigrum*; 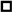 for *S. melongena*; ◆ for *S. tuberosum*). Red circles indicate the ToBRFV-infected source plant, placed at different positions. White circles represent initially virus-free plants of five species. (**B**–**F**) Bar charts showing the virus load (RT-qPCR Ct values) in tomato (**B**), pepper (**C**), black nightshade (**D**), eggplant (**E**), and potato (**F**) at 15 and 30 dpi, with and without beetles. Lower Ct values indicate higher virus loads. Bars represent mean values ± SE. Different letters above bars indicate statistically significant differences (One-way ANOVA, Duncan’s test, *p* < 0.05). Note: Lower Ct values indicate higher viral loads.

In a 60 cm × 60 cm × 80 cm mesh cage, a total of 15 healthy plants (three of each species: tomato, pepper, eggplant, potato, and black nightshade) were randomly arranged without physical contact. A single ToBRFV-infected tomato plant was placed in one of three positions (corner, edge, or center-adjacent) to act as the virus source (Figure 2A). Ten non-viruliferous *H. vigintioctopunctata* adults were introduced into each cage. Control cages had identical plant arrangements but no beetles. Each positional setup was replicated three times (within three cages). Leave samples from all initially healthy plants were collected at 15 and 30 dpi for RT-qPCR analysis.

### 2.5. Analysis of Virus Retention and Localization

#### 2.5.1. Retention of Infectivity

Two experiments were conducted to assess the persistence of transmissibility.

**Figure 3:**
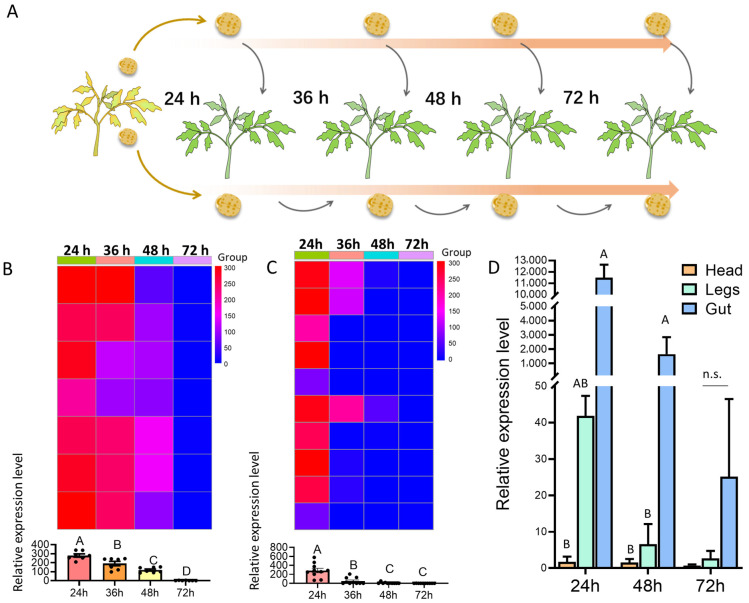
Analysis of virus transmission persistence and localization in *H. vigintioctopunctata*. (**A**) Schematic of the method for preparing viruliferous beetles. (**B**) Virus transmission efficiency of beetles starved for different durations (24, 36, 48, 72 h) after virus acquisition. (**C**) Virus transmission efficiency of beetles continuously fed on virus-free plants after acquisition. Both (**B**,**C**) show relative virus expression in recipient plants 15–30 days post-inoculation. (**D**) Relative virus load (2^−ΔΔCt^) detected in the beetle’s head, legs, and digestive tract at 24, 48, and 72 h after virus acquisition. Bars represent mean values ± SE. Different letters above bars indicate statistically significant differences (One-way ANOVA, Duncan’s test, *p* < 0.05); n.s. represents no significant difference detected among these values.

(1) Starved Beetles: Viruliferous beetles were held without food and transferred to a new healthy tomato plant at 24, 36, 48, and 72 h post-acquisition to feed (Figure 3A). Each time point had seven replicates.
(2) Continuously Fed Beetles: Viruliferous beetles were serially transferred to a new healthy tomato plant every 12 h. Transmission to plants at 24, 36, 48, and 72 h post-initial acquisition was assessed. Each time point had ten replicates.

In both experiments, plant leaf samples were collected after being treated at day 15 and day 30 for RT-qPCR.

#### 2.5.2. Viral Localization in Beetle Tissues

Viruliferous beetles were transferred onto virus-free tomato plants and cultured. At 24, 48, and 72 h post-acquisition, beetles were dissected into three parts: head, legs, and digestive tract. RNA was extracted from pooled tissues of five beetles for each time point, and the experiment was repeated three times.

### 2.6. Role of Beetle Feeding Wounds and Transmission Ability of H. vigintioctopunctata Following Varying Degrees of Feeding

To assess if feeding damage alone increases susceptibility, three treatments were established on healthy tomato plants (N= 7 replicates each): (1) application of 3 mL of infectious sap to intact leaves; (2) application of 3 mL of infectious sap to leaves previously damaged by non-viruliferous beetles; (3) positive control, where leaves were abraded with quartz sand before sap application. Plants were grown for 30 days, and adjacent leaves were collected for RT-qPCR analysis.

**Figure 4:**
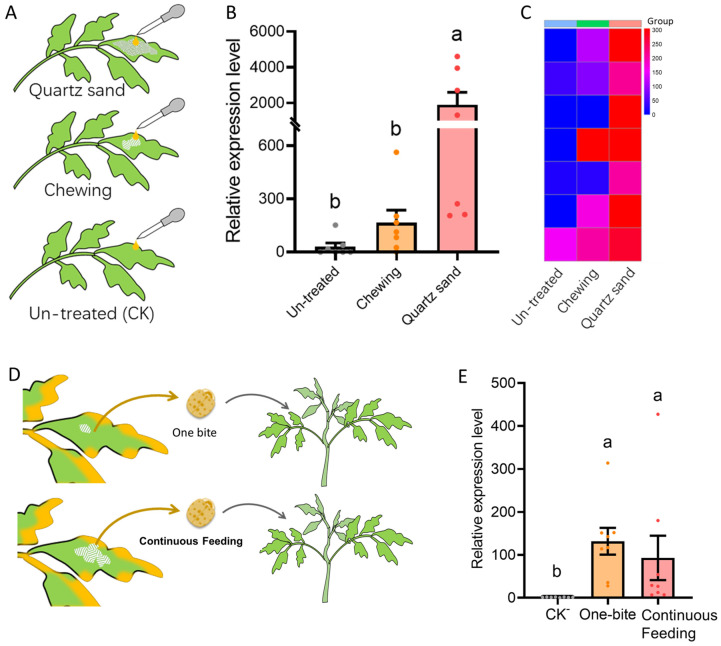
Feeding wounds created by non-viruliferous *H. vigintioctopunctata* enhance tomato plant susceptibility to ToBRFV infection (**A**–**C**) and detection of the ToBRFV transmission ability of *H. vigintioctopunctata* following varying degrees of feeding (**D**,**E**). (**A**) Schematic of the three inoculation methods: application of infectious sap to an intact leaf, a beetle-chewed leaf, or a quartz sand-abraded leaf. (**B**) Relative virus load (2^−ΔΔCt^) detected in plants 30 days post-treatment. (**C**) Heatmap showing virus load (Relative) for individual replicate samples. (**D**) Two treatments were applied: limited feeding (a single chewing hole) or continuous feeding (until natural cessation); the yellow color of the leaves in the plant schematic indicates that the plant is infected with ToBRFV and (**E**) shows relative virus load (2^−ΔΔCt^) detected among these treatments. Bars of (**B**,**E**) represent mean values ± SE. Different letters above bars indicate statistically significant differences (One-way ANOVA, Duncan’s test, *p* < 0.05).

To investigate the efficiency of ToBRFV transmission by *H. vigintioctopunctata* after different feeding durations, two treatments were implemented. In Treatment 1, beetles were allowed to create only one chewing hole on infected plants, whereas in Treatment 2, beetles fed continuously until feeding ceased naturally. Beetles from both treatments were subsequently collected and used to inoculate healthy plants. After 30 days of isolated cultivation, viral presence was detected. The experiment was repeated eight times, with virus-free beetles serving as the control group (Figure 4D).

### 2.7. Statistical Analysis

Data were tested for normality using the Shapiro–Wilk test. After normally tested, one-way analysis of variance (ANOVA) was performed, followed by Duncan’s Multiple Range Test for pairwise comparisons (*p* < 0.05) using SPSS (v. 22; IBM Corp., Armonk, NY, USA). Heatmaps were generated using the online platform Bioinformatics.com.cn (accessed on 25 May 2025) [25].

## 3. Results

### 3.1. Verification of ToBRFV Transmission by H. vigintioctopunctata

#### 3.1.1. Detection Results at 30 Days Post-Inoculation in *S. lycopersicum*

Comparative analysis between quartz sand abrasion and beetle-mediated chewing inoculation methods revealed that ToBRFV-positive signals were detectable in all treatments 30 days post-inoculation. The highest viral load was observed in the quartz sand abrasion group, followed by leaves directly chewed by *H. vigintioctopunctata*, in which the viral load was approximately 50% of the positive control. ToBRFV was also detected in non-fed leaves from the same plants, although at significantly lower levels compared to the other groups (F = 85.18, df = 3.32, *p* < 0.001; Figure 1B).

#### 3.1.2. Continuous Monitoring at 3-Day Intervals Post-Inoculation

Heatmap-based analysis of continuously sampled leaves revealed that significant ToBRFV-positive signals appeared in chewed leaves as early as 9 days post-inoculation. Unchewed leaves from the same plants began showing detectable signals by day 12. Among seven biological replicates, six exhibited high viral loads in fed leaves by day 21, and three replicates showed positive viral signals in unchewed leaves of the same plants (Figure 1D).

#### 3.1.3. Transmission of ToBRFV Among Solanaceous Plants Facilitated by *H. vigintioctopunctata*

Results from heatmaps and bar charts demonstrated that ToBRFV can be successfully transmitted from tomato to *S. nigrum* (black nightshade), *C. annuum* (pepper), and *S. melongena* (eggplant) via beetle feeding. Viral load measurements at 15 and 30 days post-treatment showed that beetle-mediated transmission to *S. nigrum* was comparable to direct mechanical inoculation with quartz sand (F = 7.088, df = 3.36, *p* = 0.007; Figure 1E). However, based on the result at day 15, transmission efficiency in *C. annuum* and *S. melongena* was significantly lower than that of the mechanical inoculation method (*C. annuum*: F = 15.86, df = 3.36, *p* < 0.001; *S. melongena*: F = 16.22, df = 3.36, *p* < 0.001; Figure 1E).

### 3.2. Cage Experiment: H. vigintioctopunctata-Facilitated Transmission of ToBRFV Among Different Solanaceous Plants

The results showed that ToBRFV was able to spread from infected tomato plants to other solanaceous species regardless of whether *H. vigintioctopunctata* was present in the system. However, the presence of the beetle significantly increased the transmission efficiency across all tested species (*S. lycopersicum*: F = 346.5, df = 3.32, *p* < 0.001; *C. annuum*: F = 54.37, df = 3.32, *p* < 0.001; *S. nigrum*: F = 186.6, df = 3.32, *p* < 0.001; *S. melongena*: F = 25.06, df = 3.32, *p* < 0.001; *S. tuberosum*: F = 226.2, df = 3.32, *p* < 0.001; Figure 2B–F).

Heatmap analysis further indicated that, in the absence of beetles, the transmission efficiency in *C. annuum* (Figure 2C) and *S. nigrum* (Figure 2D) was influenced by the spatial placement of infected tomato plants within the cage. This positional effect was notably reduced following the introduction of *H. vigintioctopunctata*, suggesting that beetle movement contributes to homogenizing transmission risk across host plants.

### 3.3. Analysis of Sustained Virus Transmission and Viral Localization in H. vigintioctopunctata

#### 3.3.1. Sustained Transmission Capacity of ToBRFV by *H. vigintioctopunctata*

Virus transmission assays conducted at 24, 36, 48, and 72 h post-virus acquisition revealed that plants exposed to viruliferous beetles at 24 h exhibited the highest viral loads 30 days post-inoculation. Viral loads declined significantly with increasing intervals, and the differences among the treatment groups were statistically significant (F = 53.01, df = 3.24, *p* < 0.001; Figure 3B). Only weak ToBRFV-positive signals were detected in the 72 h group.

In the continuous feeding group, beetles maintained high transmission efficiency at 24 and 36 h post-exposure. However, viral loads detected in plants exposed at 48 and 72 h were significantly reduced (F = 23.16, df = 3.36, *p* < 0.001; Figure 3C), suggesting a gradual decline in transmission risk over time.

#### 3.3.2. Viral Localization in Different Body Parts of *H. vigintioctopunctata*

The distribution of ToBRFV in different body parts of viruliferous beetles varied with time. In the 24 h and 48 h treatment groups, the viral load detected in the digestive tract was significantly higher than that in the legs and head (24 h: F = 47.69, df = 2.8, *p* < 0.001; 48 h: F = 99.47, df = 2.8, *p* < 0.001; Figure 3D). In the 72 h group, the digestive tract still showed the highest viral loads, followed by legs and head, although the differences were not statistically significant across tissues (F = 1.952, df = 2.8, *p* = 0.2223; Figure 3D). These results suggest that the digestive tract is the primary reservoir of ToBRFV within *H. vigintioctopunctata*, while virus presence in external structures diminishes over time.

### 3.4. Risk Assessment of Feeding Wounds Created by Virus-Free H. vigintioctopunctata

Results from the inoculation experiments showed that viral loads in plants directly treated with infectious sap were much lower than in the quartz sand abrasion group, with viral loads reaching only 1/60 of the latter after 30 days. In plants where virus-free *H. vigintioctopunctata* created feeding wounds before sap application, the mean viral load was higher than that in the direct sap inoculation group—approximately five times that of the latter—yet showed no statistically significant difference, and remained substantially lower than that in the quartz sand abrasion group (F = 6.455, df = 2.18, *p* < 0.05; Figure 4B,C).

### 3.5. ToBRFV Transmission Ability of H. vigintioctopunctata Following Varying Degrees of Feedings

The results show that a single bite from a viruliferous beetle can successfully transmit ToBRFV. Data indicated that beetles from both feeding treatments effectively transmitted ToBRFV, with no significant difference in the viral load of inoculated plants 30 days post-inoculation (F = 3.669, df = 2.21, *p* < 0.001; Figure 4E).

## 4. Discussion

This study demonstrates that the Hadda beetle, *Henosepilachna vigintioctopunctata*, is an effective mechanical vector that can amplify the spread of Tomato brown rugose fruit virus, also supporting previous conclusions regarding the role of *H. vigintioctopunctata* in facilitating virus dissemination [20,21]. Our findings confirm that the transmission is non-persistent and mechanical, a mode consistent with the known biology of tobamoviruses, which are characterized by highly stable virions that do not require specific biological interactions for transmission [1]. This non-specific mechanical transmission mode mediated by polyphagous chewing pests suggests that, even in the absence of specific biological interactions, widely distributed native pests may serve as crucial ecological bridges facilitating the rapid adaptation of invasive viruses to new environments.

The mechanism of transmission is clearly mechanical rather than biological. Several lines of evidence support this conclusion. First, the retention of infectivity is short, declining significantly after 48 h. This contrasts with semi-persistent or persistent transmission modes, which involve longer retention times and, in some cases, a latent period as the virus circulates within the vector [17]. Second, there is no evidence of a latent period, which is characteristic of circulative viruses. Third, the localization of the virus primarily in the digestive tract reflects the ingestion of infected plant material, not a systemic infection of the insect vector. The virus detected on the head and legs likely represents surface contamination that diminishes over time. This pattern is distinct from other known forms of beetle transmission, such as the semi-persistent transmission of certain viruses that involves specific binding sites in the foregut [26,27]. Therefore, ToBRFV transmission by *H. vigintioctopunctata* likely occurs via contaminated mouthparts and/or regurgitant during the feeding process. The lack of food storage function in the mouthparts may be an important reason for the low detection levels of the virus in this part. Nonetheless, this bite-based transmission still poses significant risks for virus spread, as a single bite from a viruliferous beetle can successfully transmit ToBRFV. The feces of *H. vigintioctopunctata* may potentially carry the virus, although we have not detected it in the samples to date (results were negative, with no valid Ct values in RT-qPCR). However, this potential for virus transmission cannot be ruled out.

The primary contribution of this research lies in its elucidation of the beetle’s epidemiological significance as a transmission amplifier. The cage experiment provides a compelling illustration of this role. The baseline level of transmission observed in the absence of beetles reflects the inherent stability and high mechanical transmissibility of ToBRFV [12]. However, the introduction of beetles dramatically increased both the number of infected plants and the viral load within them. The results here indicate that beetle activity carries the potential risk of accelerating the spread of an epidemic in fields. This amplification occurs through two primary pathways: direct vectoring of the virus from infected to healthy plants, and indirect facilitation of infection by creating feeding wounds that serve as entry points for the virus from other sources.

Furthermore, the beetle’s mobility and host range give it the potential to mediate virus spread at a landscape level. The 48 h retention window for infectivity is crucial; combined with the beetle’s flight capacity (Figure S1), it provides a potential mechanism for virus movement between fields and from unmanaged areas into cultivated crops. While laboratory flight mill data may not directly translate to field dispersal distances, they indicate a capacity for movement over several kilometers within the infectious window, potentially connecting disparate host populations. The demonstrated ability of *H. vigintioctopunctata* to transmit ToBRFV among different crops (tomato, pepper, eggplant) makes Solanaceae weeds act as a potential bridge and reservoir for the transmission and storage of the virus among different crops. This allows the virus to persist in the agricultural landscape, moving from weeds back to newly planted crops and circumventing control measures like crop rotation. However, we acknowledge this approach has limitations. The use of a single reference gene and the direct comparison of Ct values across different species provide an estimation of virus presence and relative abundance rather than a precise, absolute quantification. Future studies should employ absolute quantification using a standard curve or normalization with multiple validated reference genes for enhanced accuracy. Consequently, field weeds serve not only as viral reservoirs but also as ‘green bridges’ that sustain beetle populations. Future management strategies must adopt a landscape ecology perspective, quantifying the overwintering population baselines and viruliferous rates of beetles in non-crop habitats surrounding farmlands to establish more effective regional containment barriers.

## 5. Conclusions

This study presents a case of a novel ecological interaction, where an invasive pathogen (ToBRFV) leverages a native, generalist insect pest to enhance its dissemination. Such interactions, which are not based on long-term co-evolution, can unpredictably alter disease dynamics and pose new challenges for agriculture. The findings underscore that the management of ToBRFV cannot rely solely on sanitation and certified seed, particularly in regions where *H. vigintioctopunctata* is prevalent. Integrated pest management (IPM) strategies that include monitoring and control of the beetle population should be considered an essential component of a comprehensive ToBRFV management program. Understanding the synergy between invasive pathogens and native generalist herbivores is critical for predicting and mitigating the risks of emerging plant diseases in a globalized agricultural system. Obviously, the current research is confined to controlled environments. In natural settings, it is of greater significance to develop effective tracking technologies for monitoring ToBRFV transmission and to validate these findings against our conclusions. Future research should focus on quantifying the contribution of beetle-mediated transmission to ToBRFV epidemics under field conditions to further refine these integrated control strategies.

## Data Availability

The original contributions presented in this study are included in the article/Supplementary Materials. Further inquiries can be directed to the corresponding author.

## Supplementary Materials

The following supporting information can be downloaded at: https://www.mdpi.com/article/10.3390/insects16121225/s1, Table S1: List of primers utilized in this study; Figure S1: Flight performance of *H. vigintioctopunctata* on a flight mill.; Figure S2: Comparison of virus transmission ability between *H. vigintioctopunctata* individuals with mouthparts removed and intact individuals.

## Abbreviations

The following abbreviations are used in this manuscript:

ToBRFV	Tomato brown rugose fruit virus
RT-qPCR	reverse transcription quantitative PCR
