# Supplementary material for: The Role of Henosepilachna vigintioctopunctata in Facilitating the Spread of Tomato Brown Rugose Fruit Virus (ToBRFV) Among Hosts
Source: Insects. 2025 Dec 3;16(12):1225. doi: 10.3390/insects16121225 (PMC12734332; doi:10.3390/insects16121225)
Supplement: Supplementary file 1 [file insects-16-01225-s001.zip › insects-3968806-supplementary.pdf]

Supplementary materials

The Role of *Henosepilachna vigintioctopunctata* in Facilitating the Spread of Tomato Brown Rugose Fruit Virus (ToBRFV) Among Hosts

Xing-Xing Wang[1], Qing-Jiang Xing[1], Chong Zhang[1], Ya-Nan Liu[1], Tong-Xian Liu[2] and Yi Zhang[1*]

1 Shandong Engineering Research Center for Environment-Friendly Agricultural Pest Managemnent, College of Plant Health and Medicine, Qingdao Agricultural University, Qingdao, China

2 Institute of Entomology, Guizhou University, Guiyang, China

Table S1. List of primers utilized in this study

Genes		Q-RT-PCR-primers
ToBRFV	F	CATGTTTGTTACGCCGCCTT
	R	CATGCATCTTCCATTGCGCT
Q-ToBRFV	F	GCAACGGTGGCTATAAGGAG
	R	GGACCATTGTAAACCGGATG
SlUBI (ref)	F	TCGTAAGGAGTGCCCTAATGCTGA
	R	CAATCGCCTCCAGCCTTGTTGTAA
HvRPL13 (ref)	F	AGCATCCTTCGCTCGTTTA
	R	TTCGACAACCTGCCATTAGG

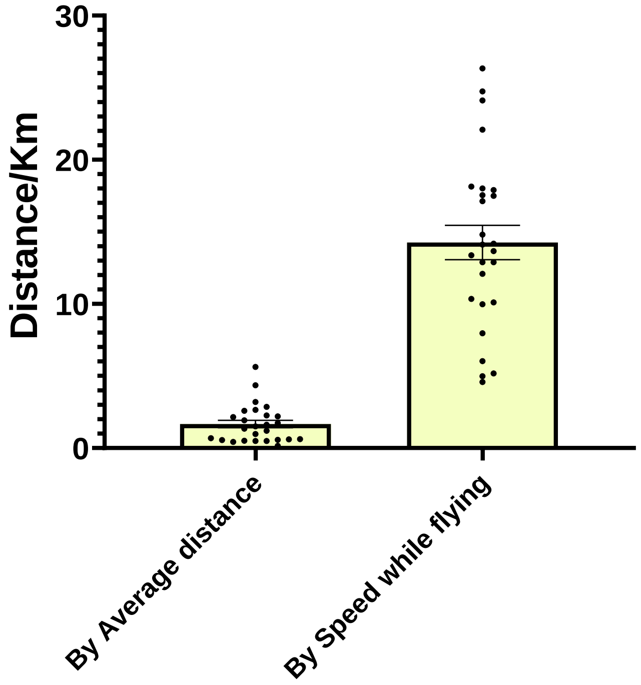

Figure S1 Flight performance of *H. vigintioctopunctata* on a flight mill. The left column represents the total flight distance recorded over a continuous 24-hour period, while the right column shows the calculated continuous 24-hour flight distance based on the average flight speed.

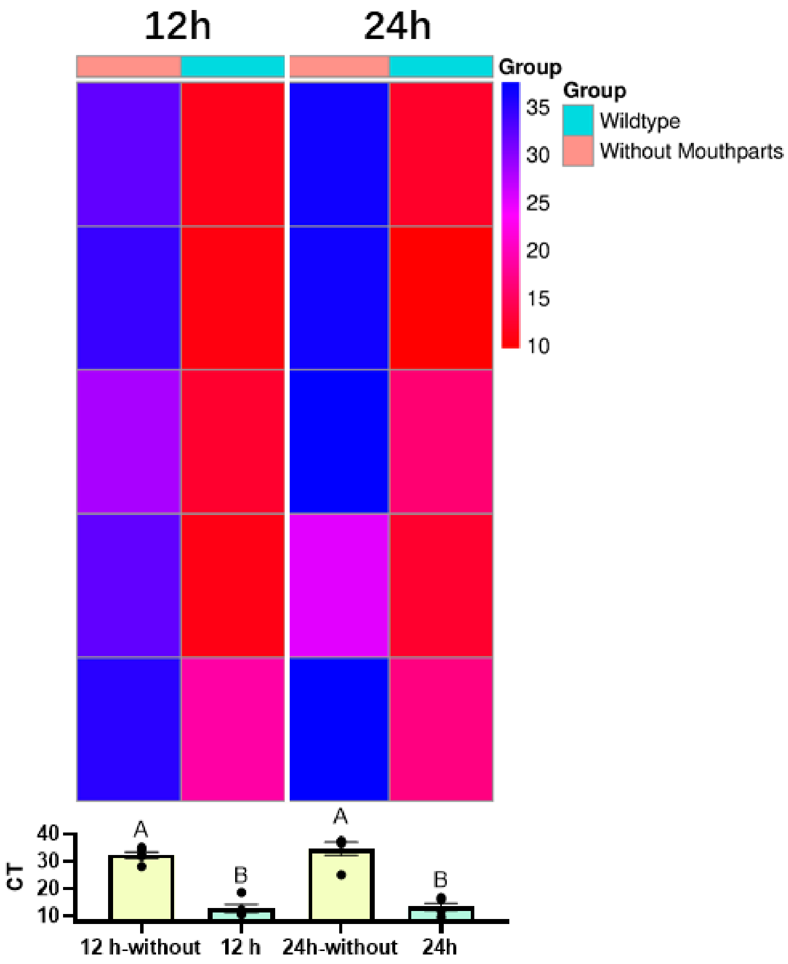

Figure S2 Comparison of virus transmission ability between *H. vigintioctopunctata* individuals with mouthparts removed and intact individuals. After feeding on ToBRFV-infected tomato leaves for 12 or 24 hours, beetles were transferred to virus-free plants for 24 hours. The plants were then cultivated in isolation for 30 days. Leaf tissues (1 cm × 1 cm) were sampled for RNA extraction and RT-qPCR analysis. The heatmap was generated based on Ct values obtained from RT-qPCR assays of individual samples. Bars represent mean values, error bars indicate standard errors (SE), and different superscript letters denote statistically significant differences as determined by one-way ANOVA ($p < 0.05$).
